# Tumor microenvironment-activated ferritin nanovector enables enhanced tumor delivery of KRAS^G12C^ inhibitors and degraders

**DOI:** 10.3389/fcell.2026.1725088

**Published:** 2026-02-25

**Authors:** Annalisa Pia Abbinantefina, Claudia Tito, Silvia Masciarelli, Giada Tisci, Siria Sortoluzzi, Pierpaolo Ceci, Elisabetta Falvo, Cécile Exertier, Francesca Troilo, Vincenzo Petrozza, Antonello Mai, Dante Rotili, Francesco Fazi, Gianni Colotti

**Affiliations:** 1 Department of Anatomical, Histological, Forensic and Orthopaedic Sciences, Section of Histology and Medical Embryology, Sapienza University of Rome, Rome, Italy; 2 Institute of Molecular Biology and Pathology, Italian National Research Council, IBPM-CNR, Rome, Italy; 3 Department of Biochemical Sciences, Sapienza University of Rome, Rome, Italy; 4 Department of Medico-Surgical Sciences and Biotechnologies, Sapienza University of Rome, Latina, Italy; 5 Department of Drug Chemistry and Technologies, Sapienza University of Rome, Rome, Italy; 6 Department of Science, “Roma Tre” University, Rome, Italy; 7 Biostructures and Biosystems National Institute (INBB), Rome, Italy; 8 Department of Wellbeing, Health and Environmental Sustainability (BeSSA), Sapienza University of Rome, Rieti, Italy

**Keywords:** Adagrasib, ferritin, KRAS, nanocarrier, PROTAC, tumor microenvironment activation

## Abstract

Mutations in RAS oncogenes (KRAS, HRAS, NRAS) are among the most common genetic alterations in human cancers. The activating KRAS^G12C^ mutation, in particular, is a key driver in a significant percentage of non-small cell lung cancer (NSCLC), pancreatic ductal adenocarcinoma (PDAC), colorectal cancer, and lung adenocarcinoma. While KRAS was long considered undruggable, the development of mutant-specific inhibitors, including covalent inhibitors targeting KRAS^G12C^ (such as Sotorasib and Adagrasib) and non-covalent inhibitors targeting KRAS^G12D^ (such as Mirati’s MRTX1133), has shown promise. These inhibitors function by binding to a shallow pocket between the switch-I and switch-II elements, locking KRAS in its inactive GDP-bound state. However, concerns exist regarding the efficacy and the development of resistance to Sotorasib and Adagrasib through mechanisms like secondary mutations, KRAS overexpression, and KRAS downstream pathway activation. To overcome these limitations, we developed a novel, stimuli-sensitive, tumor microenvironment-activated, ferritin-derived nanomedicine platform, named The-05. This platform, previously shown to effectively enhance payload biodistribution, plasma half-life, and reduce off-target effects in various tumors, is reported here to: 1) encapsulate high amounts of the KRAS^G12C^ inhibitor Adagrasib and the PROTAC degrader LC-2; 2) to achieve efficient intracellular delivery *in vitro*, once activated by matrix metalloproteases MMP-2 and MMP-9. In cellular models of KRAS-mutated NSCLC and PDAC, this nanoplatform achieved comparable or superior therapeutic outcomes with respect to the individual drugs. This study provides a compelling proof-of-concept for the *in vitro* delivery of KRAS^G12C^ mutant-specific inhibitors and degraders to human tumors through a tumor microenvironment-activated nanomedicine approach and lays the groundwork for future studies in physiologically relevant models to assess TME-specific activation and tumor selectivity.

## Introduction

Rat sarcoma (RAS) oncogenes (KRAS, HRAS, and NRAS) are among the most frequently mutated genes in human cancers. Activating KRAS G12 mutations are particularly critical drivers of malignancy, strongly associated with poor prognosis and found in approximately 15% of all human cancers. Their prevalence is even higher in specific cancer types, including pancreatic ductal adenocarcinoma (PDAC), colorectal cancer (CRC), lung adenocarcinoma (LA), and non-small cell lung cancer (NSCLC) (Thein et al., 2021; Herdeis et al., 2021; Mendiratta et al., 2021; Sinkala, 2023).

The most common KRAS mutations are G12D, G12V, and G12C. For instance, KRAS mutations are found in 90% of pancreatic cancers, with G12D or G12V present in two-thirds of PDAC and half of CRC cases. KRAS^G12C^ is the predominant KRAS mutation in NSCLC, and also occurs in LA (13%), CRC (3%), and 1%–3% of PDAC, endometrial, bladder, and ovarian cancers (Thein et al., 2021).

**FIGURE 1:**
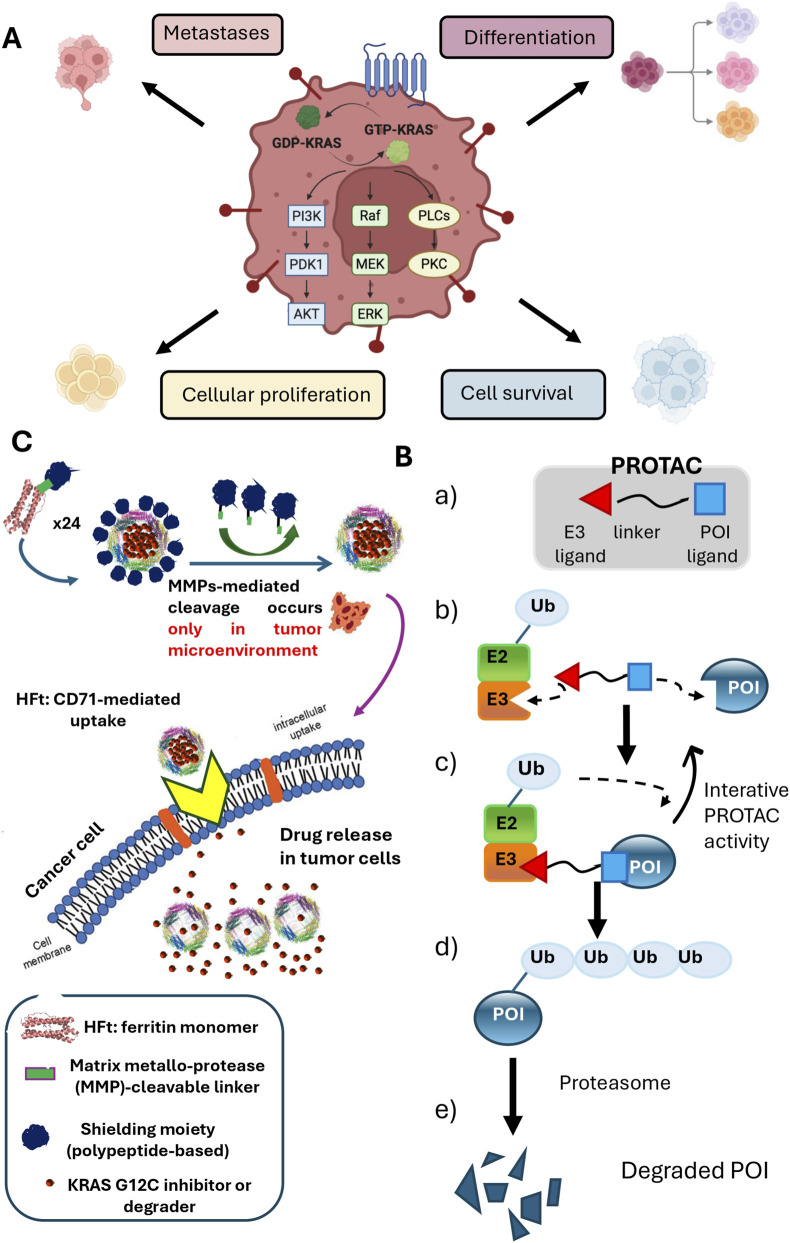
Molecular mechanisms of KRAS, PROTACs and The-05. **(A)** KRAS as a Molecular Switch and its Role in Cancer. KRAS functions as a guanosine diphosphate (GDP)/triphosphate (GTP) binary switch, which is mainly determined by regulatory guanine nucleotide exchange factors (GEFs) and GTPase-activating proteins (GAPs). KRAS controls important signal transduction from activated membrane receptors to intracellular molecules to nucleus, and particularly the PI3K-PDK1-AKT pathway, the RAF-MEK-ERK pathway, the PLC-PKC pathway and the RALGDS-RAL pathway. These pathways control cellular proliferation, survival, differentiation and angiogenesis. Mutated KRAS (in particular, KRAS mutations G12C, G12D and G12V) is permanently activated, and leads to uncontrolled signal transduction, uncontrolled cellular proliferation, survival, differentiation and angiogenesis, that lead to tumor development and metastasis. **(B)** Mechanism of action of a PROTAC. PROTACs are heterobifunctional molecules designed to induce the ubiquitination and subsequent degradation of specific proteins of interest (POIs). A PROTAC consists of three key components: a moiety that binds to an E3 ubiquitin ligase, a linker and a moiety that binds to the POI **(a)** PROTACs hijack the ubiquitin-proteasome system (UPS). The UPS is a highly conserved cellular mechanism for degrading proteins. It involves three main enzymes: Ubiquitin-activating enzyme (E1), Ubiquitin-conjugating enzyme (E2), and Ubiquitin ligase (E3). E1 activates free ubiquitin (Ub), which is then transferred to E2. E3 ligases are responsible for specifically recognizing substrates and facilitating the transfer of Ub from E2 to a lysine residue (Lys) on the substrate. Repeated ubiquitination forms a poly-ubiquitin chain, targeting the substrate for degradation by the 26S proteasome. PROTACs work by bringing the POI into close proximity with the E2-E3 complex **(b)** This trimeric complex formation facilitates the transfer of ubiquitins to the POI **(c)** The resulting poly-ubiquitinated POIs **(d)** are then rapidly degraded by the proteasome **(e)** A key feature of PROTACs is their catalytic, event-driven mechanism of action: a single PROTAC molecule can induce the degradation of multiple POI molecules, as it is released to bind another POI after inducing degradation. **(C)** Mechanism of Action of The-05 Nanocarrier. The The-05 polypeptide is engineered with an N-terminal shielding PASE moiety (blue), a matrix metalloprotease (MMP)-cleavable linker (green), and a C-terminal human ferritin heavy chain (HFt, orange). These components fold into a The-05 monomer. In the presence of the drug (either Adagrasib or the LC-2 PROTAC), and using a simple pH-dependent association-dissociation protocol, these monomers self-assemble into a shielded The-05 24-mer. The drug is encapsulated within the internal cavity of this stimuli-sensitive protein, which is then ready for administration. *In vivo*, within the tumor microenvironment (TME), the abundant MMPs cleave the PASE shield, leading to its release. The de-shielded The-05 is subsequently internalized by tumor cells via the highly expressed Transferrin Receptor 1 (TfR1, CD71). Once inside the tumor cell, the encapsulated drug is rapidly released, where it can then exert its inhibitory effect on KRAS.

The KRAS protein functions as a molecular switch, cycling between an inactive guanosine diphosphate (GDP)-bound state and an active guanosine triphosphate (GTP)-bound state. Its activity is tightly regulated by guanine nucleotide exchange factors (GEFs) and GTPase-activating proteins (GAPs), which control the binding and hydrolysis of GTP, respectively. Upon growth factor receptor signaling, particularly through EGFR, KRAS in its active GTP-bound state interacts with effector proteins such as RAF and PI3Kα via its switch I and switch II elements. This interaction activates downstream signaling pathways, including the RAF-MEK-ERK and PI3Kα-AKT-mTOR pathways, ultimately promoting cell proliferation, survival, and growth (Thein et al., 2021) (Figure 1A).

However, G12 mutations disrupt KRAS’s intrinsic GTPase activity, leading to constitutive activation independent of external growth factor signals. This effectively locks KRAS in its GTP-bound state, resulting in uncontrolled cell growth and tumorigenesis.

For decades, the high frequency of KRAS mutations and its pivotal role in maintaining the transformed phenotype fueled intense research into KRAS-targeting compounds. Yet, KRAS was long considered “undruggable” due to its picomolar affinity for GTP, the absence of suitable hydrophobic pockets, and the lack of known allosteric regulatory sites. This paradigm shifted dramatically with the pioneering work of Shokat and colleagues (Ostrem et al., 2013), which led to the development of mutant-specific inhibitors. These include covalent inhibitors targeting KRAS^G12C^ (e.g., Sotorasib and Adagrasib, the latter marketed as Krazati® and receiving FDA breakthrough therapy designation for NSCLC in 2021) and high-affinity (single-digit nanomolar) non-covalent inhibitors targeting KRAS^G12D^ (e.g., Mirati’s MRTX1133). These inhibitors bind to a shallow pocket situated between the switch I and switch II elements, effectively trapping mutated KRAS in its inactive GDP-bound state, disrupting both switch I and switch II, and impairing its interaction with RAF (Fell et al., 2020; Wang et al., 2022). This breakthrough has led to a surge of newly designed KRAS inhibitors and degraders in recent years (Wei et al., 2024; Ash et al., 2024).

However, despite their initial promise, a significant challenge with existing KRAS inhibitors like Sotorasib and Adagrasib is the rapid development of resistance in many patients, often within months of treatment initiation. This resistance arises from diverse mechanisms, categorized as either on-target or off-target. On-target resistance mechanisms include secondary KRAS mutations, KRAS overexpression, and KRAS reactivation (due to interactions with EGFR and/or Aurora kinase A). Off-target resistance mechanisms involve the activation of alternative downstream pathways, epigenetic changes, transcriptional reprogramming, alterations in the tumor microenvironment (TME), or epithelial-to-mesenchymal transition (Xue et al., 2020; Awad et al., 2021; Tanaka et al., 2021; Ash et al., 2024; Dilly et al., 2024; Kumarasamy et al., 2024).

Beyond inhibition, protein degradation offers a promising avenue to overcome resistance mechanisms, especially those linked to KRAS overexpression or reactivation. KRAS undergoes various post-translational modifications, including ubiquitination at sites such as Lys104, Lys117, and Lys147 (Herdeis et al., 2021). These ubiquitination sites can be exploited by PROteolysis TArgeting Chimeras (PROTACs) (Sakamoto et al., 2001). PROTACs are heterobifunctional small molecules designed to induce selective intracellular proteolysis. Unlike traditional enzyme inhibitors, PROTACs consist of two covalently linked protein-binding moieties: an E3 ubiquitin ligase-engaging ligand and a ligand that binds to the target protein of interest (POI) (Figure 1B). By recruiting an E3 ligase (e.g., von Hippel–Lindau protein (VHL), Cereblon (CRBN), or inhibitors of apoptosis proteins (IAPs)) to the target protein, PROTACs induce polyubiquitination and subsequent proteasomal degradation of the POI. This mechanism is particularly advantageous because PROTACs only require selective binding to their targets, rather than inhibiting enzymatic activity, opening up opportunities to re-purpose previously ineffective inhibitor molecules. Furthermore, KRAS chemical degradation could directly address several key resistance mechanisms observed with KRAS inhibitors, such as secondary mutations, KRAS overexpression, and KRAS reactivation. For instance, the VHL-based PROTAC, LC-2, which utilizes Adagrasib as a KRAS^G12C^ warhead, has demonstrated rapid and sustained KRAS degradation and impaired pERK downstream signaling for up to 72 h in several KRAS^G12C^-mutated cell lines, achieving maximum degradation values of up to 90% (Bond et al., 2020). Given the challenges of acquired resistance and the urgent need for more effective therapies, novel strategies are required to improve the efficacy of KRAS inhibitors and degraders. Enhancing the selectivity of drug delivery to tumors is crucial to increase potency, reduce off-target effects, and mitigate the development of resistance (Di Stefano, 2023).

In this work, we present an innovative nanomedicine platform based on the human ferritin protein, designed to address these challenges. We utilized a family of stable, non-toxic, and stimuli-sensitive ferritin-derived nanocarriers, amenable to molecular biology modifications, to significantly enhance payload biodistribution, extend plasma half-life, and minimize off-target effects. These nanocarriers have already shown remarkable efficacy against various tumors and possess the unique ability to encapsulate substantial amounts of mutation-specific KRAS inhibitors and degraders, delivering them selectively and efficiently to tumor cells.

Specifically, we cloned and expressed several human ferritin (HFt) variants, each composed of 24 monomers consisting of a ferritin moiety and an inert shielding polypeptide rich in proline (P), alanine (A), serine (S) and glutamate (E) residues (PASE), linked by a matrix metalloprotease (MMP)-cleavable linker. We employed the previously reported variant The-05 to encapsulate high amounts of the KRAS^G12C^-specific inhibitor Adagrasib and the degrader PROTAC LC-2 (Figure 1C). The The-05 nanocarrier forms a cage capable of incorporating numerous drug molecules (up to 150, depending on the variant sequence) within its cavity via a simple pH-shift protocol. This shielded nanocarrier remains inactive during circulation, exhibiting high stability, extended plasma half-life, minimal drug leakage, and low unspecific cell binding (off-target effects) until MMP-mediated cleavage occurs exclusively within the tumor microenvironment (TME), where MMPs are highly expressed (Fracasso et al., 2016; Falvo et al., 2018; Falvo et al., 2020; Falvo et al., 2021; Fracasso et al., 2023; Marrocco et al., 2024) (Figure 1C). Upon selective shield release in the TME, The-05 is effectively and rapidly internalized by virtually all types of cancer cells via the CD71 receptor (Transferrin/ferritin receptor). CD71 is highly expressed by rapidly growing tumor cells, including metastatic loci, due to their substantial iron demand. This selective uptake ensures that drug-containing HFt is efficiently delivered to tumor cells, where the payload is rapidly released and can bind its target. We have previously demonstrated the efficacy of these stimuli-sensitive nanocarriers, particularly the latest version incorporating anthracyclines or the topoisomerase I inhibitor Genz-664282, in xenograft murine models of pancreatic and breast cancers, an orthotopic pancreatic tumor model, and a pancreatic PDX model. In all these studies, efficient and specific drug delivery to the tumor was observed, with no apparent off-target toxicities, resulting in strong and long-lasting reductions in tumor growth or volume, and a marked increase in post-therapy survival (Falvo et al., 2021; Conti et al., 2021; Fracasso et al., 2023; Marrocco et al., 2024).

In the present study, we leverage our innovative, stimuli-sensitive, ferritin-derived nanomedicine platform to encapsulate high amounts of the KRAS^G12C^-specific inhibitor Adagrasib and the degrader PROTAC LC-2, and to deliver them specifically and with high efficiency to tumor cells in cellular models of KRAS-mutated NSCLC and PDAC.

## Materials and methods

### Protein production and characterization

The nanocarrier platform used in this study belongs to the family of PASE-shielded, MMP-responsive human ferritin variants (The-05 series). These constructs include an external shielding PASE domain, a matrix-metalloprotease (MMP-2/9)-cleavable linker, and a CD71-targeting ferritin core, conferring both stability in circulation and conditional activation within the tumor microenvironment. The production and full characterization of The-05 is reported in Fracasso et al. (2016), Falvo et al. (2018); Falvo et al. (2020). Briefly, a construct with the expressing sequence of the heavy chain of human ferritin (HFt), where the negatively charged glutamic acid residue (Glu) was introduced in place of four native HFt residues, namely Lys53, Lys71, Thr135, and Lys143, followed by a MMP sequence (PLGLAG), which is recognized as a proteolytic cleavage site by matrix metallo-proteinases such as MMP-2 and MMP-9, and by an outer shield-forming PASE sequence (ASPAAPAPASPAEPAPSAPAASPAAPAPASPAEPAPSAPA), was cloned in a pET-11a expression vector. The recombinant protein The-05 was expressed in *E. coli*, purified and quantified as previously described (Falvo et al., 2016; Falvo et al., 2018; Falvo et al., 2020).

### Compounds

Adagrasib (MRTX849) was obtained by Selleck Chem (HPLC: 99.76% purity).

The MRTX849-based, VHL recruiting PROTAC LC-2 was prepared following a procedure adapted from the original report by Crews and co-workers (see Supplementary Figure S1) (Bond et al., 2020). The commercially available 1-tert-butyl 4-ethyl 3-oxopiperidine-1,4-dicarboxylate (2) was reacted with commercial 2-methylisothiourea in dry MeOH in the presence of NaOMe at room temperature to provide *tert*-butyl 4-hydroxy-2-methylsulfanyl-6,8-dihydro-5H-pyrido [3,4-*d*]pyrimidine-7-carboxylate (3). This latter was treated with Tf2O in dry DCM in the presence of DIPEA to give the intermediate *tert*-butyl 2-(methylthio)-4-(((trifluoromethyl)sulfonyl)oxy)-5,8-dihydropyrido [3,4-d]pyrimidine-7(6H)-carboxylate (4) that was then heated at 100 °C in dry DMF in the presence of benzyl-(2S)-2-(cyanomethyl)piperazine-1-carboxylate1 and DIPEA to furnish the *tert*-butyl (S)-4-(4-((benzyloxy)carbonyl)-3-(cyanomethyl)piperazin-1-yl)-2-(methylthio)-5,8-dihydropyrido [3,4-d]pyrimidine-7(6H)-carboxylate (5). After deprotection with TFA in dry DCM in the presence of TIS at room temperature the resulting benzyl (S)-2-(cyanomethyl)-4-(2-(methylthio)-5,6,7,8-tetrahydropyrido [3,4-d]pyrimidin-4-yl)piperazine-1-carboxylate was treated with commercially available 1-bromo-8-chloro-naphthalene in the presence of Pd_2_ (dba)_3_, RuPhos, and Cs_2_CO_3_ in dry toluene at 100 °C to provide the intermediate benzyl (S)-4-(7-(8-chloronaphthalen-1-yl)-2-(methylthio)-5,6,7,8-tetrahydropyrido [3,4-d]pyrimidin-4-yl)-2-(cyanomethyl)piperazine-1-carboxylate (6). After oxidation with m-CPBA in dry DCM at 0 °C the corresponding sulphoxide (7) was reacted with freshly prepared *tert*-butyl (S)-3-(3-(2-(hydroxymethyl)pyrrolidin-1-yl)propoxy)propanoate1 in dry toluene in the presence of t-BuONa at 0 °C to give the intermediate benzyl (S)-4-(2-(((S)-1-(3-(3-(tert-butoxy)-3-oxopropoxy)propyl)pyrrolidin-2-yl)methoxy)-7-(8-chloronaphthalen-1-yl)-5,6,7,8-tetrahydropyrido [3,4-d]pyrimidin-4-yl)-2-(cyanomethyl)piperazine-1-carboxylate (8). After hydrogenolysis by stirring under H_2_ atmosphere in the presence of Pd/C in dry MeOH at room temperature, the subsequent coupling with commercially available 2-fluoroacrylic acid in the presence of HATU and DIPEA in dry DMF at room temperature gave the intermediate tert-butyl 3-(3-((S)-2-(((7-(8-chloronaphthalen-1-yl)-4-((S)-3-(cyanomethyl)-4-(2-fluoroacryloyl)piperazin-1-yl)-5,6,7,8-tetrahydropyrido [3,4-d]pyrimidin-2-yl)oxy)methyl)pyrrolidin-1-yl)propoxy)propanoate (9). After ester deprotection with TFA in dry DCM at room temperature, the resulting acid was finally coupled with the commercial (1R)-1-[(2S,4R)-4-hydroxy-2-[[4-(4-methylthiazol-5-yl)phenyl]methylcarbamoyl]pyrrolidine-1-carbonyl]-2,2-dimethyl-propyl in the presence of HATU and DIPEA to provide the desired final compound (2S,4R)-1-((S)-2-(3-(3-((S)-2-(((7-(8-chloronaphthalen-1-yl)-4-((S)-3-(cyanomethyl)-4-(2-fluoroacryloyl)piperazin-1-yl)-5,6,7,8-tetrahydropyrido [3,4-d]pyrimidin-2-yl)oxy)methyl)pyrrolidin-1-yl)propoxy)propanamido)-3,3-dimethylbutanoyl)-4-hydroxy-N-(4-(4-methylthiazol-5-yl)benzyl)pyrrolidine-2-carboxamide (LC-2, 1) (Supplementary Figure S1).

LC-2 characterization by 1H-NMR, 13C-NMR spectra, high-resolution mass spectra (HR-MS) and elemental analysis is reported in Supplementary Figure S2.

### Protein encapsulation of drug Adagrasib and LC-2 PROTAC

Adagrasib KRAS^G12C^ inhibitor and LC-2 KRAS^G12C^ PROTAC were encapsulated in The-05 using the ferritin disassembly/reassembly procedure previously described for other compounds, with a drug: protein molar ratio of 120:1 (Falvo et al., 2020). Briefly, solutions of The-05 (2 mg/mL) in H_2_Odd were incubated for 10 min at pH 3.1 (pH adjusted with HCl). Protein disassembly/reassembly was achieved by dropwise addition of NaOH to pH = 7.5. After 20 min of stirring at room temperature, the product was filtered to eliminate insoluble particles. An excess of unbound drug/PROTAC was removed using 100 kDa Amicon Ultra-15 centrifugal devices, in 10 mM Tris-HCl at pH 7.5. Finally, the solution was sterile filtered and stored at 2–8 °C in the dark.

Adagrasib and LC-2 were quantified by UV-vis spectroscopy, after extracting the drug in 0.1 N HCl, by using the calculated molar extinction coefficient ε = 9,780 Mˉ^1^ cm ˉ^1^ at 333 nm. Protein content was also determined by UV-vis spectroscopy applying the following correction for the absorbance at 280 nm: A280 nm - (A333 nm x 1.6). LC-2 PROTAC was quantified by using the calculated molar extinction coefficient ε = 7,700 Mˉ^1^ cm ˉ^1^ at 333 nm. Protein content was also determined by UV-vis spectroscopy applying the following correction for the absorbance at 280 nm: A280 nm - (A333 nm x 3.2).

Effective encapsulation and slow drug leakage was demonstrated by repeated diafiltration experiments (Tris buffer exchange experiments by repeatedly concentrating the sample and reconstituting it with a new buffer) where The-05-Adagrasib and The-05-LC-2 samples were microdialyzed with an Amicon Ultra MWCO 10 kDa microfiltration apparatus, and the extent of drug leakage into the was assessed by measuring UV-vis spectra of both retentate protein and eluate samples.

### Complex characterization

The purity of all preparations was assessed by SDS-PAGE on 15% acrylamide gels stained with GelCode Blue Safe Protein Stain (Thermofisher scientific, Milan, Italy). Size-Exclusion Chromatography (SEC) experiments were performed using a Superose 6 gel-filtration column equilibrated with PBS at pH 7.4. All samples were prepared at 1 mg/mL in filtered PBS. All the traces for SEC experiments were analyzed with QtiPlot (IONDEV SRL, Bucuresti, Romania).

Mass photometry experiments were performed using the Refeyn OneMP instrument. Samples (200 nM) were diluted to 20 nM in PBS buffer on the glass coverslips, previously assembled into sample chambers with silicone gaskets to prevent evaporation. Data were acquired for 60 s per sample and processed using DiscoverMP software to detect individual landing events and generate mass distribution. Calibration was performed using protein standards of known molecular weight (MassFerence™ P1 Calibrant) diluted in PBS buffer.

### Cell lines and treatment

Human, KRAS^G12C^-mutated, pancreatic adenocarcinoma (PDAC) MiaPaCa-2 and non-small cell lung cancer (NSCLC) Calu-1 cell lines, were maintained in DMEM medium (Gibco® Thermo Fisher Scientific) supplemented with 50 U/mL penicillin, 50 U/mL streptomycin, and 10% heat-inactivated fetal bovine serum (FBS) at 37 °C in incubator with humidified 5% CO_2_ atmosphere.

In all experiments, 3.5 × 10^5^ cells/well were seeded into 6-well dishes and after 24 h treated with Adagrasib or the LC-2 PROTAC, either alone or encapsulated in The-05 nanoparticles, or with buffer (NT) or with empty The-05 at the highest concentration used for each experiment (FT), pre-treated to simulate conditions within the Tumor Microenvironment (TME). Specifically, The-05-Adagrasib and The-05-LC-2 were pre-incubated for 30 min at 37 °C with Collagenase IV (*Clostridium* histolyticum, containing MMP-2 and MMP-9; Panbiotech, Leipzig, Germany) at a 1:1,000 M ratio to increase the levels of metalloproteases, enhancing the release of the PASE shielding moiety.

Calu-1 cells and MiaPaCa2 cells were treated with Adagrasib at final concentrations of 5 nM, 25 nM, 100 nM and 500 nM for 72 h, either alone or encapsulated in The-05 nanoparticles.

Calu-1 cells and MiaPaCa2 cells were treated with LC-2 PROTAC at final concentrations of 500 nM, 1 μM and 2 µM for 72 h either alone or encapsulated in The-05 nanoparticles.

Encapsulated drug concentrations were calculated from the The-05 nanoparticle concentration and drug loading per nanoparticle. Free and encapsulated drugs were tested at equivalent molar concentrations.

### Cell proliferation and cell death assay

For proliferation analysis, 3.5 × 10^5^ cells/well were seeded into 6-well dishes and after 24 h, treated with Adagrasib or LC-2 PROTAC, either alone or encapsulated or encapsulated in The-05 nanoparticles for 72 h. Proliferation was assessed using the Trypan Blue exclusion test. Cell death was evaluated by the propidium iodide exclusion assay and was analyzed by flow cytometry (CyAN ADP DAKO, Beckman Coulter, Brea, CA, United States).

### Lysate preparation and immunoblotting analysis

Cells were lysed in 2% SDS buffer (supplemented with 10 μL PMSF, 10 µL Na_2_VO_3_, 20 µL PIC, 1 µL Leupeptin, 1 µL NaF, 25 µL Na_2_PO_4_). Lysates were sonicated for 15 s, centrifuged at 12,000 rpm for 10 min to remove debris, and quantified using the Bradford assay (Thermo Fisher Scientific) following the manufacturer’s protocol. Western blotting was performed loading 40 µg of protein lysates onto SDS-PAGE gels, followed by transfer onto nitrocellulose membranes (Protran). Membranes were blocked with 5% milk for 1 h and incubated overnight with primary antibodies: mouse monoclonal GAPDH (1:2000, #TA802519, OriGene Technologies, Rockville, United States), rabbit monoclonal Phospho-ERK1/ERK2 (P44/P42 MAPK) (T202/Y204 #MAB-94122 Immunological Science, Italy); rabbit mono-clonal p44/42 MAPK (Erk1/2) (1:1,000 #4695 Cell Signaling, Danvers, MA, United States); rabbit polyclonal PanRAS (1:1,000 #3965, Cell Signaling Technology, Beverly, Massachusetts, United States). As secondary anti-bodies were used goat anti-mouse (1:10,000) and anti-rabbit (1:5,000) conjugated to horseradish peroxidase (Bethyl Laboratories, Montgomery, TX, United States). Protein signals were developed using ECL detection using a ChemiDoc-It Imaging System (UVP, Upland, CA) instrument.

### Statistical analysis

Statistical analyses were performed using GraphPad Prism (GraphPad Software, San Diego, CA, United States). Comparisons between groups were conducted using one-way analysis of variance (one-way ANOVA). Data are presented as average ±standard error of the mean (SEM). All experiments were performed with n = 3 independent replicates. A P value ≤0.05 was considered statistically significant.

## Results

### Encapsulation and characterization of the-05 loaded with KRAS^G12C^ inhibitor (Adagrasib) and degrader (LC-2)

We successfully encapsulated the KRAS^G12C^ inhibitor Adagrasib and the PROTAC degrader LC-2 within the internal cavity of our The-05 nanocarrier using an adapted protein disassembly/reassembly method (Falvo et al., 2020; see Methods). This process achieved an encapsulation efficiency of approximately 43 ± 10 (43% ± 23%) molecules of Adagrasib (corresponding to ∼39–43% of the initial 100–110 molecules) and 90 ± 10 (90% ± 11%) molecules of LC-2 (corresponding to ∼82–90% of the initial 100–110 molecules) per The-05 24-mer. For both compounds, the overall protein recoverywas approximately 70%. Protein concentration before and after encapsulation was determined by UV-visible spectroscopy by measuring absorbance at 280 nm. Protein recovery was calculated as the percentage of protein retained after purification relative to the initial amount, according to the following equation:

$$\text{Protein Recovery }\left(\%\right)=\left(\text{Protein}\_\text{after purification }/\text{ Protein}\_\text{initial}\right)\times 100$$

The reported value (about 70%) indicates that approximately 30% of the initial protein was lost during the disassembly/reassembly and filtration steps.

**FIGURE 2:**
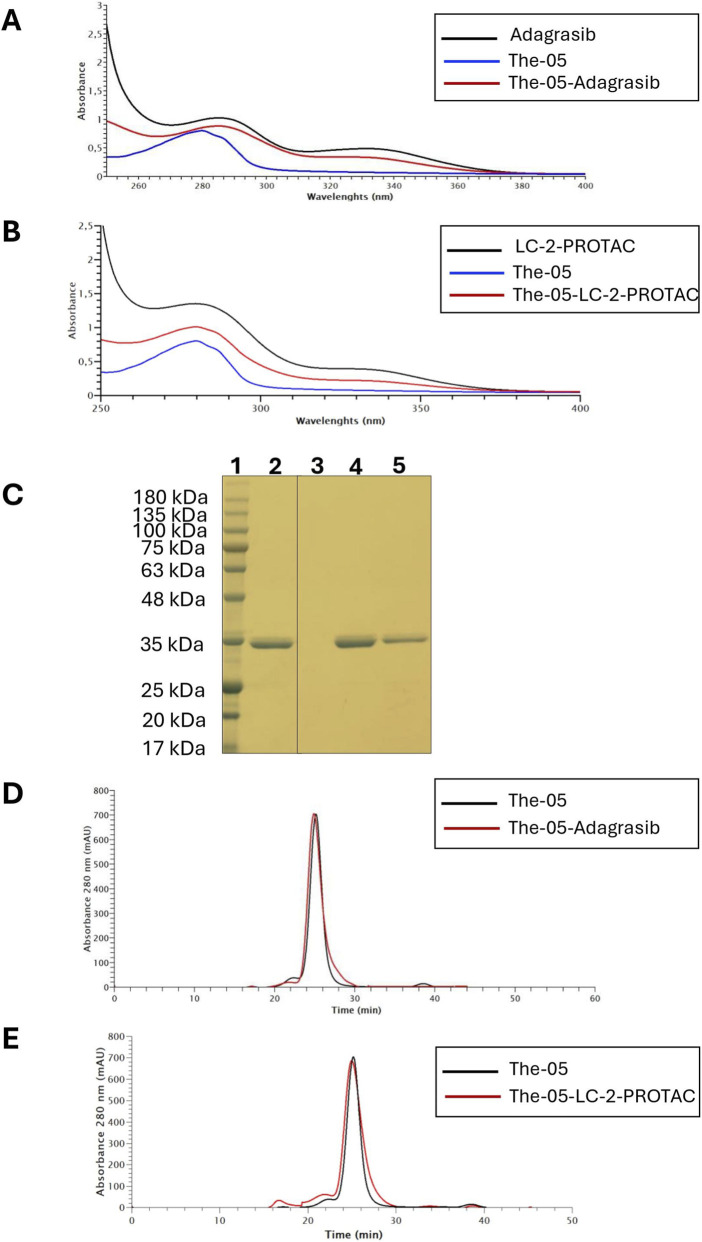
The-05 Encapsulation, Purity and Assembly. **(A)** UV spectra analysis of the individual components and the complex: The-05 nanocarrier (blue), KRAS^G12C^ inhibitor Adagrasib (black), and the The-05-Adagrasib complex (red). **(B)** UV spectra analysis of the individual components and the complex: The-05 nanocarrier (blue), PROTAC LC-2 (black), and the The-05-LC-2 complex (red). These spectra confirm the successful encapsulation of both Adagrasib and LC-2 within the The-05 nanocarrier. **(C)** SDS-PAGE analysis of The-05 constructs: Protein band migration profiles demonstrating the purity of the The-05 nanocarrier before and after drug encapsulation. Lane 1: Protein molecular weight marker; Lane 2: Purified The-05 (10 µg); Lane 4, The-05-Adagrasib complex (10 µg); Lane 5, The-05-LC-2 complex (10 µg). **(D)** Size-exclusion chromatography (SEC) profile of The-05 (black) and The-05-Adagrasib complex (red). **(E)** Size-exclusion chromatography (SEC) profile of The-05 (black) and The-05-LC-2 complex (red). SEC profiles confirm the monodispersity of the nanocarrier and complexes, and the absence of significant changes after drug encapsulation, further supporting the structural integrity of The-05.

Representative UV spectra confirmed the successful complex formation for both The-05-Adagrasib (Figure 2A) and The-05-LC-2 (Figure 2B).

Further characterization by SDS-PAGE and size-exclusion chromatography (SEC) confirmed the purity, monodispersity, and structural integrity of the nanocarrier:drug complexes. SDS-PAGE revealed that The-05 migrated as a single, pure band at approximately 35 kDa (Figure 2C). Importantly, no significant differences were observed in protein structure or assembly after drug encapsulation, indicating that the encapsulation process does not compromise the overall integrity of the The-05 construct (Figures 2D,E). Moreover, SEC profiling showed no evidence of compound aggregation or drug leakage after storing the complexes at −20 °C for 6 months, suggesting excellent long-term stability. Long-term stability studies of the drug-loaded The-05 constructs are currently ongoing. While the present work includes a 6-month stability evaluation demonstrating drug encapsulation, with no detectable aggregation or drug leakage (Supplementary Figure S3), extended storage and stress-test assessments will be addressed in future studies to further support the translational development of this nanocarrier system.

To exclude nonspecific surface adsorption, we performed a control incubation of The-05 with each drug at neutral pH, without acidic disassembly/reassembly (data not shown). Under these conditions, loading efficiency remained below 10%, confirming that significant incorporation occurs only after cage opening and that the observed loading values reflect true internal encapsulation.

Mass photometry experiments were performed using a Refeyn OneMP instrument to evaluate the susceptibility of the PASE sequence of The-05-Adagrasib to metalloproteases of the TME. The-05-Adagrasib samples (20 nM) in PBS buffer, both untreated and pre-treated to simulate conditions within the Tumor Microenvironment (TME). Specifically, The-05-Adagrasib was pre-incubated for 30 min at 37 °C with Collagenase IV (*Clostridium* histolyticum, containing MMP-2 and MMP-9; Panbiotech, Leipzig, Germany) at a 1:1 M ratio. This step, essential to trigger the release of the PASE shielding moiety, effectively releases PASE, preparing the nanovector for effective internalization via CD71 (Supplementary Figure S4).

### Efficacy of the-05-encapsulated KRAS inhibitors in cellular models of KRAS^G12C^-Mutated NSCLC and PDAC

**FIGURE 3:**
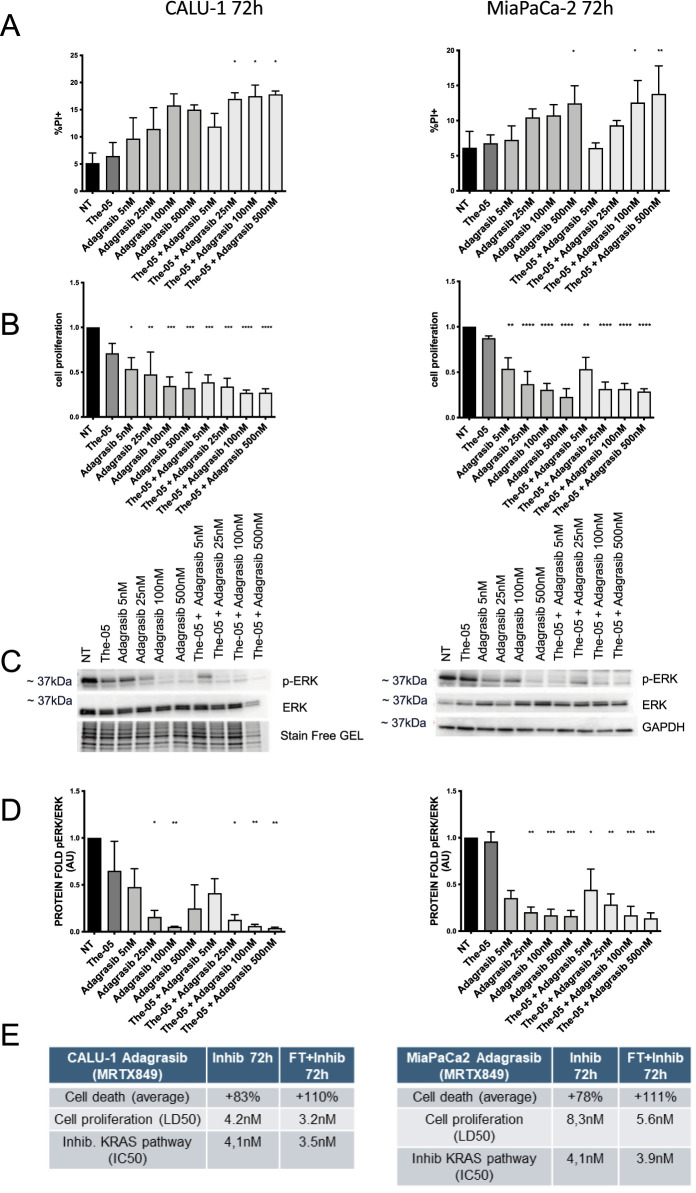
Efficacy of The-05-Encapsulated Adagrasib in KRAS^G12C^-Mutated Cancer Cells. Dose-dependent effects of free Adagrasib and The-05-Adagrasib on Calu-1 (NSCLC, left panels) and MiaPaCa-2 (PDAC, right panels) cell cultures. **(A)** cell death and **(B)** cell proliferation 72 h after administration of (from left to right): 1. Buffer (NT); 2. Empty The-05 (FT); 3–6. Free Adagrasib at 5, 25, 100, and 500 nM; 7–10. The-05-Adagrasib at equivalent Adagrasib concentrations (5, 25, 100, and 500 nM). **(C,D)**: KRAS pathway inhibition (phosphorylated ERK/total ERK ratio) after 72 h of administration: (Top) Representative western blots; (Bottom) Quantitative analysis of phosphorylated ERK/total ERK ratio for the same treatment groups as in **(A)**. Reduced pERK levels indicate effective KRAS pathway inhibition. **(E)**: Summary of drug LD50 and KRAS IC50 value., Data fitted from **(A,B,D)**, comparing the half-maximal lethal dose (LD50) for cell death and the half-maximal inhibitory concentration (IC50) for KRAS pathway inhibition for both free and The-05-encapsulated Adagrasib. Data are presented as average ±standard error of the mean (SEM). Statistical significance was assessed using one-way analysis of variance (ANOVA). Statistical significance: *p < 0.05; **p < 0.01; ***p < 0.001; ****p < 0.0001. All experiments were performed using n = 3 biological replicates.

We investigated the efficacy of both free and The-05-encapsulated KRAS^G12C^ inhibitors and degraders in Calu-1 (NSCLC) and MiaPaCa-2 (PDAC) cell lines, both harboring the KRAS^G12C^ mutation. First, we confirmed high expression levels of the CD71 receptor (TfR1) in both cell lines (Supplementary Figure S5), indicating that The-05 can be readily internalized by these cancer cells via CD71-mediated uptake. Accordingly, after 72 h The-05-Adagrasib treatment showed a similar dose-dependent effect to Adagrasib free in Calu-1 (left panels) and MiaPaCa-2 (right panels) cells (Figure 3A). Specifically, in Calu-1 cells, we observed a statistically significant increase in cell death following The-05-Adagrasib treatment at concentrations of 25 nM, 100 nM, and 500 nM, compared to both untreated cells and cells treated with Adagrasib alone (Figures 3A,E). Accordingly, the proliferation assay showed that The-05-Adagrasib treatment at higher doses (100 nM and 500 nM) was slightly more effective than Adagrasib alone, leading to a 74% reduction in cell proliferation compared to 66% with Adagrasib (Figures 3B,E). Similarly, KRAS pathway inhibition, assessed by the phosphorylated ERK/total ERK ratio after 72 h of treatment (Figures 3C–E), confirmed the enhanced efficacy of Adagrasib when delivered via The-05 compared to the free drug formulation. In parallel, the analysis of The-05-Adagrasib treatment on cell death, cell proliferation, and KRAS pathway inhibition in MiaPaCa-2 cell lines showed a comparable effect between free Adagrasib and Adagrasib encapsulated in the The-05 nanocarrier, suggesting a lower responsiveness of MiaPaCa-2 cells to The-05 delivery system compared to Calu-1 cells. These findings highlight the therapeutic potency of Adagrasib when delivered by our nanocarrier platform in the NSCLC and PDAC cell lines.

### Efficacy and KRAS degradation with the-05-encapsulated KRAS degraders in cellular models

**FIGURE 4:**
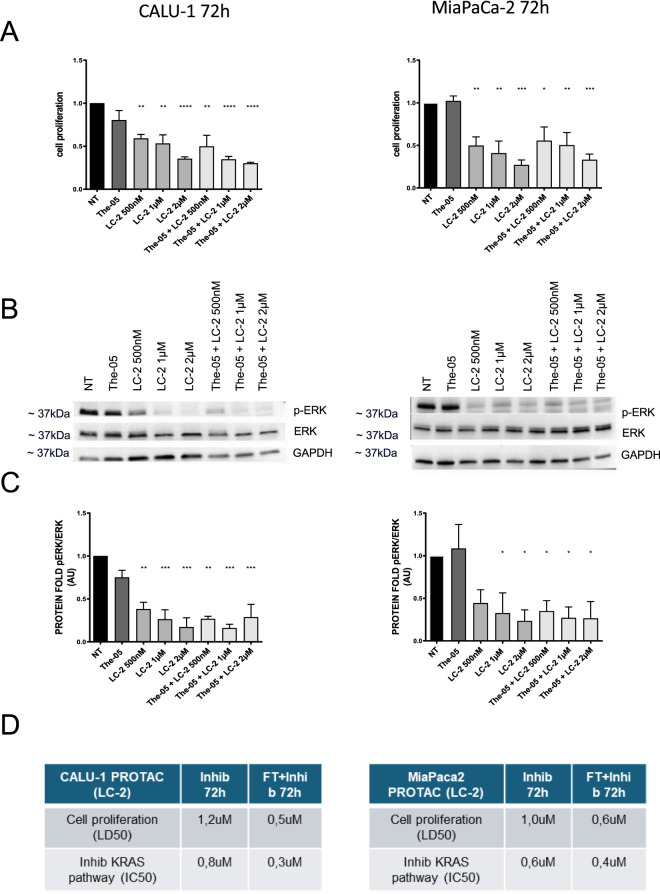
Efficacy of The-05-Encapsulated PROTAC LC-2 in KRAS^G12C^-Mutated Cancer Cells. Dose-dependent effects of free PROTAC LC-2 and The-05-LC-2 on Calu-1 (NSCLC, left panels) and MiaPaCa-2 (PDAC, right panels) cell cultures. **(A)** Cell proliferation after 72 h of administration: Comparison of cell proliferation with (from left to right): 1. Buffer (NT); 2. Empty The-05 (FT); 3–5. Free PROTAC LC-2 at 500 nM, 1 μM, and 2 μM; 6–8. The-05-LC-2 at equivalent LC-2 concentrations (500 nM, 1 μM, and 2 µM). **(B,C)** KRAS pathway inhibition (phosphorylated ERK/total ERK ratio) after 72 h of administration: (Top) Representative western blots; (Bottom) Quantitative analysis of phosphorylated ERK/total ERK ratio for the same treatment groups as in **(A)**. Reduced pERK levels indicate effective KRAS pathway inhibition. **(D)** Summary of PROTAC LD50 and KRAS IC50 values: Data fitted from **(A,C)**, comparing the half-maximal lethal dose (LD50) for cell death and the half-maximal inhibitory concentration (IC50) for KRAS pathway inhibition for both free and The-05-encapsulated LC-2. Data are presented as average ±standard error of the mean (SEM). Statistical significance: *p < 0.05; **p < 0.01; ***p < 0.001; ****p < 0.0001. Statistical significance was assessed using one-way analysis of variance (ANOVA). Statistical significance: *p < 0.05; **p < 0.01; ***p < 0.001; ****p < 0.0001. All experiments were performed using n = 3 biological replicates.

Similar enhancements were observed when testing the PROTAC degrader LC-2, both free and encapsulated in The-05, in Calu-1 and MiaPaCa-2 cell lines (Figure 4). As for Adagrasib, Calu-1 cells displayed greater sensitivity to the activity of The-05 LC-2 compared to MiaPaCa-2 cells. The analysis of cell proliferation and KRAS pathway inhibition in Calu-1 showed the increase in drug activity for The-05-LC-2 compared to free LC-2 at doses of 500 nM and 1 µM. Specifically, at 500 nM and 1 μM, cell proliferation decreased by 38% and 37% with LC-2, and by 53% and 66% with The-05-LC-2, respectively, compared to untreated cells (Figure 4A, right panel). In parallel, ERK phosphorylation was reduced by 61% and 73% with LC-2, and by 73% and 83% with The-05-LC-2, respectively (Figures 4B,C, right panel). In contrast, although The-05-LC-2 also showed statistically significant effects in MiaPaCa-2 cells, its activity was comparable to that of free LC-2 (Figures 4A,B–C, left panel), suggesting differential sensitivity of cancer cell lines to the nanocarrier-based delivery system. The fitted values of cell proliferation LD50 (dose at which 50% reduction in cell proliferation is observed) is 0.5 µM for The-05-LC-2, compared to 1.2 µM for free LC-2 in Calu-1, while it is 0.6 µM for The-05-LC-2, compared to 1.0 µM for free LC-2 in MiaPaCa cells; the fitted values of KRAS IC50 (dose at which 50% inhibition of KRAS pathway is observed) is 0.3 µM for The-05-LC-2, compared to 0.8 µM for free LC-2 in Calu-1, while it is 0.4 µM for The-05-LC-2, compared to 0.6 µM for free LC-2 in MiaPaCa cells (Figure 4D).

KRAS degradation upon treatment was also measured for The-05-LC-2 compared to free LC-2 at doses of 500 nM, 1 μM and 2 µM in Calu-1. 6 h after treatment with 500 nM, 1 µM and 2 μM, KRAS levels decreased by 62%, 80% and 84% with LC-2, and by 71%, 79% and 80% with The-05-LC-2, respectively, compared to untreated cells (Supplementary Figure S6). The fitted DC50 (concentration at which 50% KRAS degradation is observed) is 0.2 µM for The-05-LC-2, compared to 0.4 µM for free LC-2.

## Discussion

KRAS plays a central role in signal transduction, and KRAS mutations are strongly implicated in tumor initiation and development. Despite nearly 40 years of research, effectively targeting KRAS in cancers has remained a formidable challenge. Recent breakthroughs in targeting specific KRAS mutations, such as G12C, have opened new avenues for precision oncology. However, challenges remain, primarily concerning therapeutic efficacy and the pervasive development of resistance. As aptly stated by Herdeis et al. (2021), achieving effective KRAS inhibition while overcoming resistance would be akin to “stopping the beating heart of cancer.”

In this study, we employed our innovative, stimuli-sensitive, ferritin-derived nanomedicine platform to encapsulate high amounts of the KRAS^G12C^-specific inhibitor Adagrasib and the PROTAC degrader LC-2. The nanocarrier platform used in this study belongs to the family of PASE-shielded, MMP-responsive human ferritin variants (The-05 series). These constructs include an external shielding PASE domain, a matrix-metalloprotease (MMP-2/9)-cleavable linker, and a CD71-targeting ferritin core, conferring both stability in circulation and conditional activation within the tumor microenvironment. Our characterization revealed that the resulting nanocarriers were highly pure, monodisperse, and maintained excellent stability over time.

When administered to cellular models of KRAS-mutated NSCLC and PDAC, these nanocarriers facilitated the specific and efficient delivery of the KRAS^G12C^ inhibitor and PROTAC to tumor cells. This targeted delivery achieved comparable or, more often, superior outcomes compared to the administration of the individual free drugs. This enhanced efficacy was consistently demonstrated across various assays, including cell proliferation, KRAS pathway inhibition, and, for the PROTAC, direct KRAS degradation. To note, the effect of PROTAC LC-2 is always lower, at the same concentration, than that of the inhibitor from which it is derived; this, already described in the work of Bond and collaborators (2020), probably derives from the mechanism of action of PROTAC, which is less efficient than the direct one of the inhibitor.

Our The-05 nanocarrier was deliberately designed to remain shielded and inactive during systemic circulation. This design ensures high stability, a prolonged plasma half-life, minimal drug leakage, and reduced off-target effects. Its activation is precisely controlled by matrix metalloproteases (MMPs), which are abundantly expressed in the tumor microenvironment (TME), but at much lower levels in healthy tissues (Fracasso et al., 2016; Falvo et al., 2018; Falvo et al., 2020; Falvo et al., 2021; Fracasso et al., 2023; Marrocco et al., 2024).

In interpreting the findings of this work, it is important to distinguish between the *in vitro* setting used here and the *in vivo* tumor microenvironment where The-05 activation normally occurs. *In vitro*, functional delivery is observed due to the addition of collagenase (MMP-2 and MMP-9), mimicking the TME activity. Therefore, while these results provide a strong proof-of-concept, the tumor-selective delivery of The-05 remains to be validated under physiologically relevant conditions. Previous *in vivo* studies with similar stimuli-sensitive ferritin nanocarriers have demonstrated efficient and specific drug internalization via the CD71 (transferrin/ferritin) receptor (Falvo et al., 2020; Falvo et al., 2021; Fracasso et al., 2023; Marrocco et al., 2024). This receptor is highly expressed across numerous tumor types, including metastatic sites, reflecting the elevated iron demand of rapidly proliferating tumor cells. This mechanism ensures that drug-loaded ferritin is selectively incorporated by tumor cells, where the payload is rapidly released and can bind its target. Our extensive previous studies, utilizing these stimuli-sensitive nanocarriers (including recent iterations with anthracyclines or the topoisomerase I inhibitor Genz-664282) in xenograft murine models of pancreatic and breast cancers, orthotopic pancreatic tumors, and pancreatic PDX models, have consistently demonstrated efficient and specific drug delivery, negligible off-target toxicities, significant and sustained tumor growth reduction, and notably improved post-therapy survival (Falvo et al., 2021; Conti et al., 2021; Fracasso et al., 2023; Marrocco et al., 2024).

This study is intended as an *in vitro* proof-of-concept demonstrating that KRAS-targeted small molecules can be successfully encapsulated within the The-05 platform while preserving biological activity. Comprehensive *in vivo* pharmacokinetic, biodistribution, and safety evaluations will be reported separately. Given these compelling results, we anticipate that TME-activated The-05 loaded with KRAS inhibitors or PROTACs will exhibit even more pronounced enhanced performance *in vivo* compared to the *in vitro* results presented here. This nanomedicine platform represents a highly promising therapeutic strategy against PDAC and NSCLC tumors harboring the KRAS^G12C^ mutation, offering a potential path to overcome current limitations in efficacy and resistance. Acknowledging these limitations, we are currently conducting further *in vivo* studies to validate this hypothesis and explore the potential of this TME-responsive nanomedicine platform for KRAS^G12C^-mutated cancers.

## Data Availability

The raw data supporting the conclusions of this article will be made available by the authors, without undue reservation.
