# Supplementary material for: Tumor microenvironment-activated ferritin nanovector enables enhanced tumor delivery of KRASG12C inhibitors and degraders
Source: Front Cell Dev Biol. 2026 Feb 25;14:1725088. doi: 10.3389/fcell.2026.1725088 (PMC12976860; doi:10.3389/fcell.2026.1725088)
Supplement: Supplementary file 1 [file DataSheet2.pdf]

Supplementary Figure 2

Characterization of LC-2: NMR, HR-MS, Elemental Analysis

^{1}H-NMR and ^{13}C-NMR spectra for **LC-2** were recorded on a Bruker Avance III HD600 NMR spectrometer (600 MHz for ^{1}H-NMR and 151 MHz for ^{13}C-NMR). The values of chemical shifts (δ) are reported in p.p.m. High-resolution mass spectra (HR-MS) was performed using a Q Exactive UHMR Hybrid Quadrupole-Orbitrap mass spectrometer (Thermo Fisher). Elemental analysis was used to determine the purity of LC-2, which was >95%; the analytical results were within ±0.40% of the theoretical values.

^{1}H-NMR (600 MHz, Acetone-d6) δ 8.82 (s, 1H), 7.85 (t, J = 7.9 Hz, 2H), 7.70 (dd, J = 8.0, 3.9 Hz, 1H), 7.55 (dd, J = 7.3, 3.6 Hz, 1H), 7.53 – 7.48 (m, 1H), 7.45 (dd, J = 7.8, 4.2 Hz, 2H), 7.43 – 7.39 (m, 1H), 7.38 – 7.33 (m, 3H), 7.27 – 7.23 (m, 1H), 5.33 – 5.20 (m, 2H), 4.64 (dd, J = 9.3, 1.6 Hz, 1H), 4.60 – 4.48 (m, 3H), 4.39 – 4.27 (m, 3H), 4.22 – 4.08 (m, 2H), 3.84 – 3.78 (m, 2H), 3.78 – 3.70 (m, 2H), 3.67 – 3.53 (m, 3H), 3.53 – 3.43 (m, 3H), 3.37 (dd, J = 13.8, 3.8 Hz, 1H), 3.34 – 3.24 (m, 2H), 3.23 – 3.12 (m, 4H), 3.11 – 2.96 (m, 4H), 2.80 – 2.75 (m, 1H), 2.71 – 2.65 (m, 1H), 2.48 – 2.39 (m, 4H), 2.39 – 2.32 (m, 1H), 2.26 – 2.18 (m, 1H), 2.18 – 2.11 (m, 1H), 2.11 – 2.06 (m, 1H), 1.91 – 1.82 (m, 1H), 1.79 – 1.61 (m, 6H), 0.96 (s, 9H).

^{13}C-NMR (151 MHz, Acetone-d6) δ 172.62, 171.40, 171.34, 170.96, 167.67, 166.06, 163.77, 157.79, 151.29, 149.68, 149.41, 149.18, 140.54, 138.50, 132.34, 131.35, 130.61, 130.53, 129.43 (2C), 128.77, 127.78 (2C), 126.75, 126.69, 119.91, 118.28, 110.17, 77.33, 70.71, 69.85, 69.83, 67.74, 63.39, 60.60, 59.98, 59.75, 57.59, 57.46, 54.94, 53.21, 51.27, 51.21, 43.20, 38.44, 38.43, 37.41, 36.50, 32.70, 26.41 (3C), 24.05, 23.39, 20.89, 19.43, 16.41, 14.56, 14.41.

HR-MS [$C_{59}H_{71}ClFN_{11}O_{7}S$] Calculated: 1131,4931; Observed: 1131,4936.

Elemental Analysis: calculated C, 62.56; H, 6.32; N, 13.60; S, 2.83; F, 1.68; Cl, 3.13; found C, 62.66; H, 6.36; N, 13.55; S, 2.78; F, 1.70; Cl, 3.10.
