# Supplementary material for: Tumor microenvironment-activated ferritin nanovector enables enhanced tumor delivery of KRASG12C inhibitors and degraders
Source: Front Cell Dev Biol. 2026 Feb 25;14:1725088. doi: 10.3389/fcell.2026.1725088 (PMC12976860; doi:10.3389/fcell.2026.1725088)

Supplementary Figure 4

Mass photometry analysis by Refeyn.
Mass photometry measurements were performed using a Refeyn OneMP instrument. Individual landing events were converted into molecular mass values using calibration standards of known molecular weight. The resulting mass distribution is shown as a histogram, where peaks correspond to distinct oligomeric species. Gaussian fitting was used to determine the mean molecular mass and relative population of each species. One major population is observed at approximately 600 kDa for The-05-Adagrasib (orange) and 500 kDa for The-05-Adagrasib treated with collagenase (blue). Events below ~60 kDa, including buffer background, were excluded from the analysis, as indicated by the presence of a corresponding negative-mass population. Measurements were carried out at 20 nanomolar protein concentrations in PBS buffer.

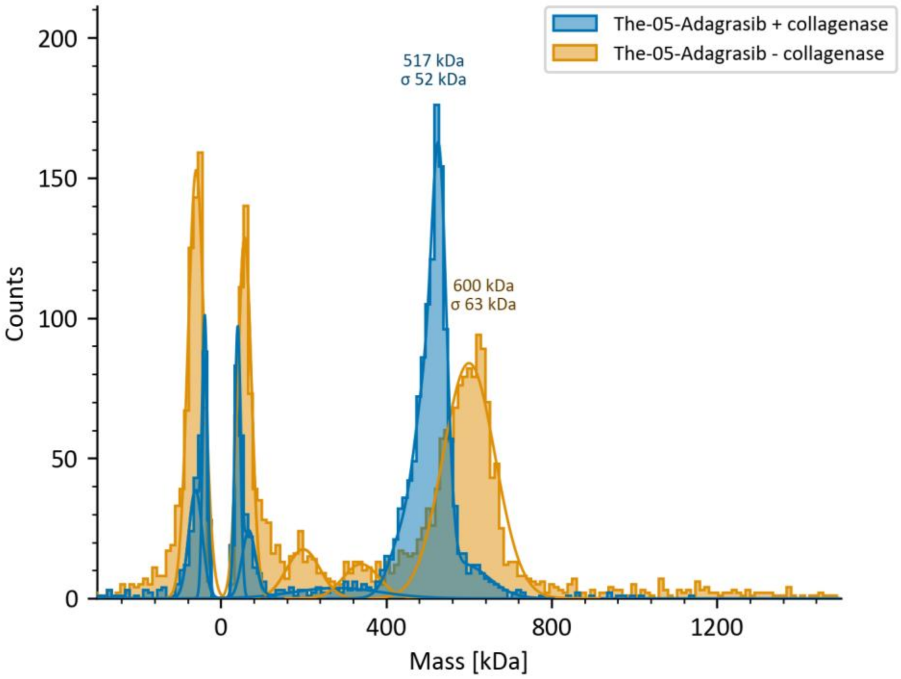

Supplement: Supplementary file 2 [file DataSheet4.pdf]
