# Supplementary material for: Tumor microenvironment-activated ferritin nanovector enables enhanced tumor delivery of KRASG12C inhibitors and degraders
Source: Front Cell Dev Biol. 2026 Feb 25;14:1725088. doi: 10.3389/fcell.2026.1725088 (PMC12976860; doi:10.3389/fcell.2026.1725088)
Supplement: Supplementary file 3 [file DataSheet6.pdf]

Supplementary Figure 6

KRAS degradation (measured as KRAS level/GAPDH ratio), 6h after treatment in Calu-1 cells.

From left to right: 1. Buffer (NT); 2. Empty The-05 (FT); 3. 500 nM LC2 PROTAC; 4. 1 µM LC2 PROTAC; 5. 2 µM LC2 PROTAC; 6. 500 nM The-05-LC2; 7. 1 µM The05-LC2; 8. 2 µM The05-LC2.

(Top: representative western blots; bottom: PanRAS quantification from PanRAS/GAPDH ratios).

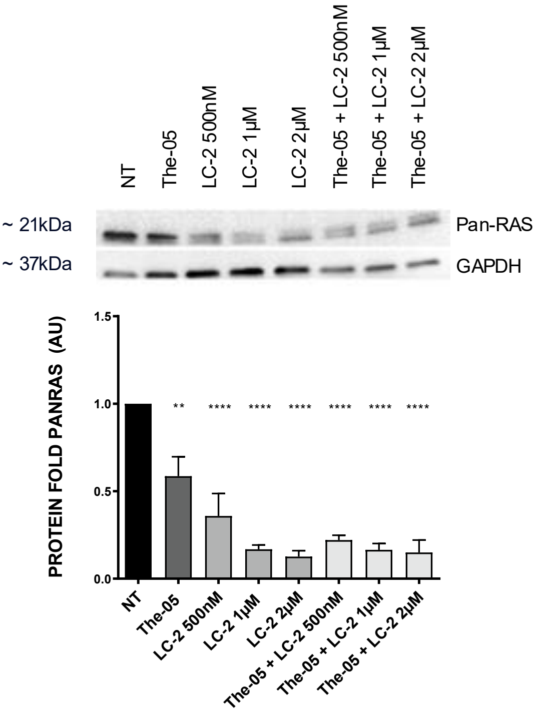

CALU-1 PROTAC (LC-2)	LC-2 degrader	The-05-LC-2 degrader
Degradation KRAS (DC50)	0,4uM	0,2uM
