# Supplementary material for: Tumor microenvironment-activated ferritin nanovector enables enhanced tumor delivery of KRASG12C inhibitors and degraders
Source: Front Cell Dev Biol. 2026 Feb 25;14:1725088. doi: 10.3389/fcell.2026.1725088 (PMC12976860; doi:10.3389/fcell.2026.1725088)

Supplementary Figure 3

Characterization of The-05-Adagrasib:
6-month stability evaluation demonstrating drug encapsulation, with little detectable aggregation and drug leakage.

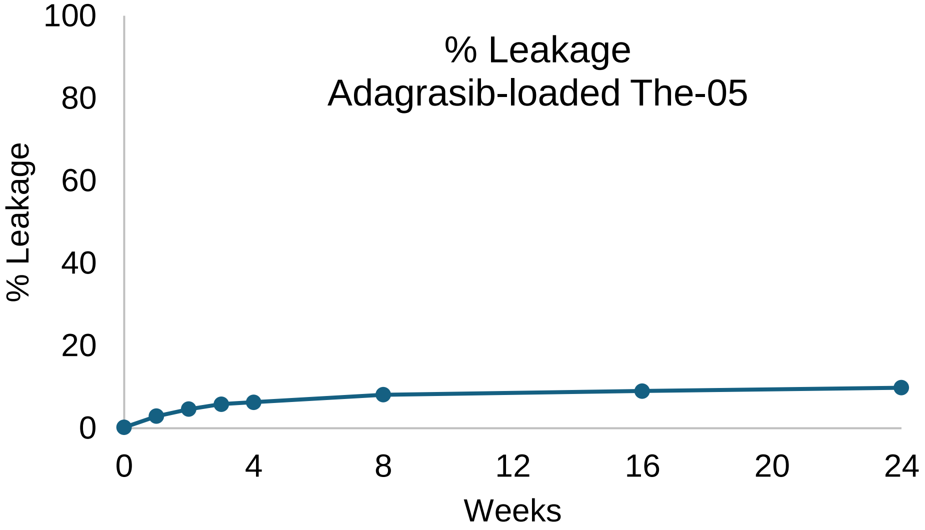

Supplement: Supplementary file 4 [file DataSheet3.pdf]
