# Supplementary material for: Tumor microenvironment-activated ferritin nanovector enables enhanced tumor delivery of KRASG12C inhibitors and degraders
Source: Front Cell Dev Biol. 2026 Feb 25;14:1725088. doi: 10.3389/fcell.2026.1725088 (PMC12976860; doi:10.3389/fcell.2026.1725088)

Supplementary Figure 1
Synthesis scheme of the PROTAC LC-2.

Scheme 1
Reagents and conditions: a) 2-methylisothiourea, MeONa, dry MeOH, 20°C, 16h; b) Tf2O, TEA, dry DCM, N2, 0°C to 25°C, 16h; c) benzyl-(2S)-2-(cyanomethyl)piperazine-1-carboxylate, DIPEA, dry DMF, N2, 100°C, 1h; d) TFA, dry DCM, TIS, N2, 20°C, 1h; e) 1-bromo-8-chloro-naphtalene, Pd2(dba)3, RuPhos, Cs2CO3, toluene, N2, 100°C, 12h; f) m-CPBA, DCM, 0°C, 1h; g) tert-butyl (S)-3-(3-(2-(hydroxymehtyl)pyrrolidin-1-yl)propoxy)propanoate), tBuONa, dry toluene, 0°C, 30 min; h) NH3 7N in MeOH, Pd/C, MeOH, H2, 20°C, 4h; i) 2-fluoroacrylic acid, HATU, DIPEA, dry DMF, r.t., 1h; l) TFA, dry DCM, r.t., 30 min.; m) (1R)-1-[(2S,4R)-4-hydroxy-2-[(4-(4-methylthiazol-5-yl)phenyl]methylcarbamoyl] pyrrolidine-1-carbonyl]-2,2-dimethyl-propyl, HATU, DIPEA, dry DMF, r.t., 1h.

Synthesis scheme.

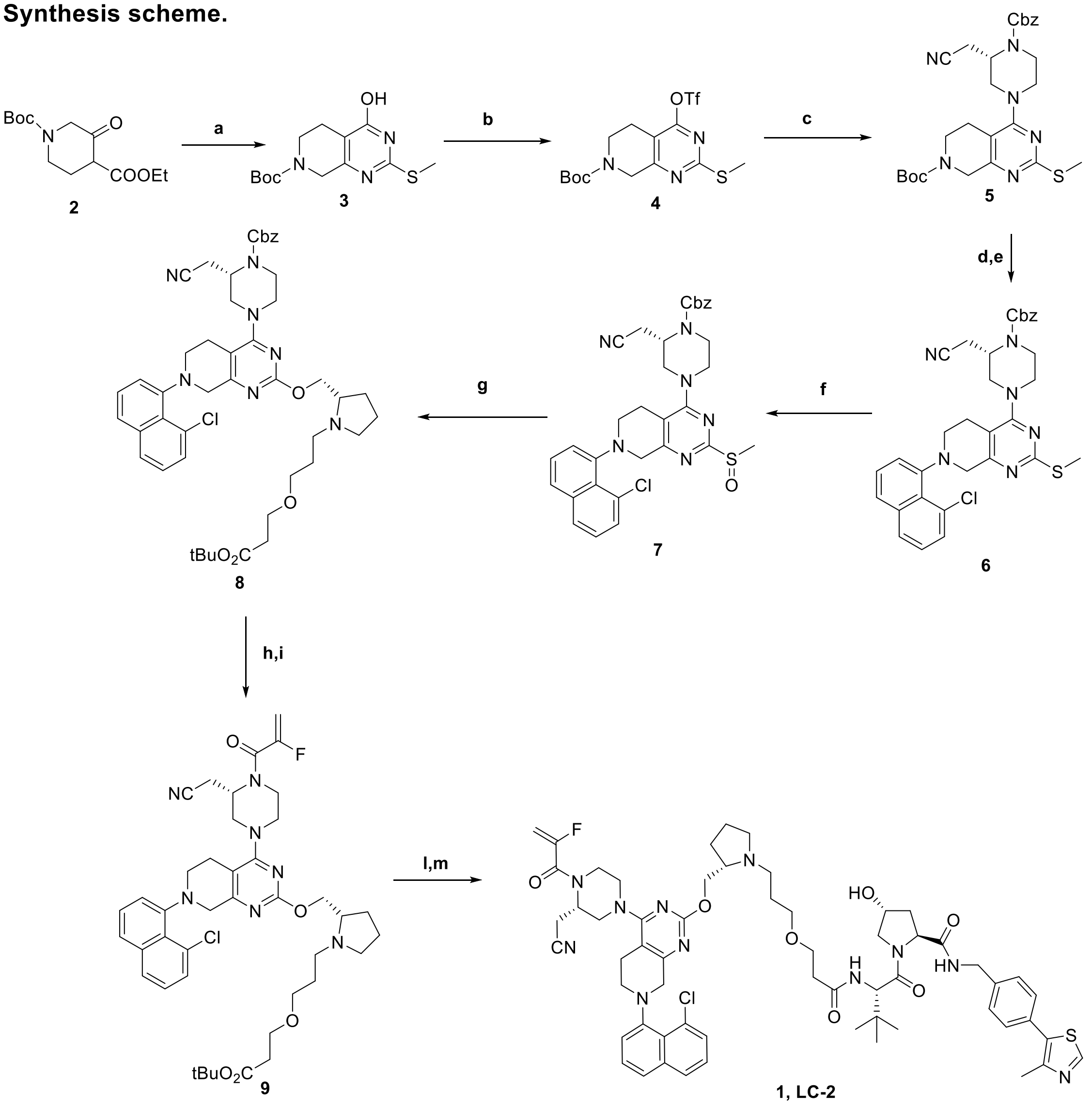

Supplement: Supplementary file 5 [file DataSheet1.pdf]
