# Supplementary material for: Tumor microenvironment-activated ferritin nanovector enables enhanced tumor delivery of KRASG12C inhibitors and degraders
Source: Front Cell Dev Biol. 2026 Feb 25;14:1725088. doi: 10.3389/fcell.2026.1725088 (PMC12976860; doi:10.3389/fcell.2026.1725088)

Supplementary Figure 5

Expression of TfR1 (CD71) in Calu-1 cell lines (non-small lung carcinoma) and MiaPaCa2 cell line (pancreatic ductal adenocarcinoma).

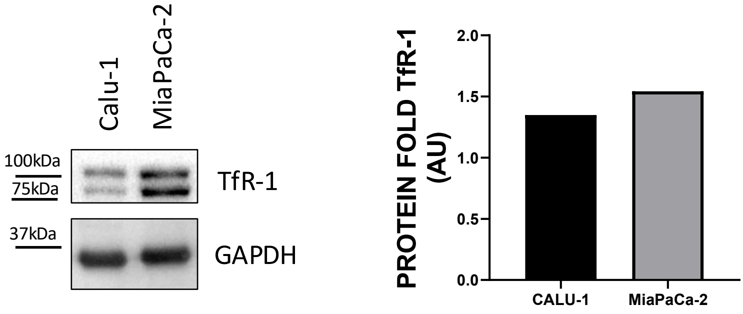

Supplement: Supplementary file 6 [file DataSheet5.pdf]
